# Defining estimands in anaesthesia clinical trials: a scoping review and framework

**DOI:** 10.1016/j.bja.2026.03.026

**Published:** 2026-04-20

**Authors:** Gethmini A. Siriwardena, Sarah Luu, Rukshala K. Gunaratne, Julie A. Simpson, Kate Leslie, Sabine Braat, Anurika P. De Silva

**Affiliations:** 1 Centre for Epidemiology and Biostatistics, Melbourne School of Population and Global Health, University of Melbourne, Melbourne, VIC, Australia; 2 Department of Critical Care, Melbourne Medical School, University of Melbourne, Melbourne, VIC, Australia; 3 Department of Anaesthesia, Austin Health, Melbourne, VIC, Australia; 4 Nuffield Department of Medicine, University of Oxford, Oxford, UK; 5 Methods and Implementation Support for Clinical and Health (MISCH) Research Hub, University of Melbourne, Melbourne, VIC, Australia; 6 Department of Anaesthesia and Pain Management, Royal Melbourne Hospital, Melbourne, VIC, Australia

**Keywords:** anaesthesia clinical trials, clinical trial design, estimand framework, ICH E9(R1), intercurrent events, perioperative medicine, randomised controlled trial, scoping review

## Abstract

**Background:**

Intercurrent events, such as treatment switching and rescue medication, commonly occur in anaesthesia trials, and can affect the occurrence or interpretation of primary outcomes. The estimand framework provides strategies for handling these events, aligning trial objectives with outcomes.

**Methods:**

We conducted a scoping review of RCTs with ≥100 participants published from January 2020 to June 2024 in ten high-impact journals, including patients receiving anaesthesia before surgery. We examined whether estimands were reported and identified intercurrent events. A structured framework was developed by mapping intercurrent events to intervention-outcome combinations investigated across all included anaesthesia trials.

**Results:**

Among 139 included trials, none explicitly used the estimand framework, and an estimand could be inferred in only 27 (19%). The primary outcome was defined in all trials, while other attributes were often not inferable: target population (44 [32%]), treatment condition (21 [15%]), summary measure (22 [16%]) and intercurrent event handling (84 [60%]). The most common intercurrent events were not receiving the randomised intervention (64 [46%]), treatment discontinuation (38 [27%]), and treatment switching (27 [19%]). Anaesthesia-related intercurrent events included surgery cancellation (37 [27%]) and non-protocolised anaesthesia management (17 [12%]). Findings informed the development of a structured, interactive web-based tool linking intercurrent events to intervention-outcome combinations, refined by expert review.

**Conclusions:**

Use of estimands in anaesthesia trials is limited. Our tool supports structured identification and handling of intercurrent events in anaesthesia trials. Greater collaboration between statisticians and clinicians, with consistent application of the estimand framework, is needed to improve clarity and interpretability of future anaesthesia research.

**Editor’s key points**

• Intercurrent events frequently occur in anaesthesia trials and can influence interpretation of treatment effects. However, use of the estimand framework to address these events remains unclear.
• This scoping review finds that explicit use of estimands in anaesthesia trials is rare, and key estimand attributes are often incompletely reported.
• The authors propose a structured framework and interactive tool to help identify intercurrent events and support clearer specification of estimands in future anaesthesia trials.

Global investment and research activity in anaesthesia trials has expanded rapidly over the past decade, reflecting their pivotal role in advancing perioperative medicine.^1^ As the gold standard for generating robust and credible clinical evidence,^2^ RCTs in anaesthesia have delivered pivotal insights that have redefined best practice across diverse domains, including pain management, cognitive recovery, cardiopulmonary complications, and perioperative infections.^3^, ^4^, ^5^, ^6^, ^7^, ^8^ Events occurring after randomisation or treatment initiation, such as use of rescue medication, treatment discontinuation, treatment switching, or death, are common in clinical trials.^9^ These events are referred to as intercurrent events and can influence the interpretation or the existence of a trial outcome.^10^ Beyond these, anaesthesia trials encounter additional intercurrent events specific to perioperative care, including conversion from regional to general anaesthesia, surgery cancellation, unplanned ICU admission, and emergency re-operation.^11^ These context-specific events increase the complexity of anaesthesia trials, potentially leading to fundamentally different research questions than originally intended, limiting the relevance of trial findings for patients, clinicians, and policymakers.

To address these challenges, the International Council for Harmonisation of Technical Requirements for Pharmaceuticals for Human Use (ICH) released the E9(R1) addendum in 2019, introducing the estimand framework to clinical trials.^10^ This framework aligns trial objectives with the design, conduct, statistical analysis, and interpretation of results, thereby ensuring that trials answer their intended research questions. An estimand clearly defines the treatment effect of interest through five key attributes: the treatment condition(s), population, outcome variable, population-level summary measure, and strategies for handling intercurrent events. Although closely resembling the well-known Population-Intervention-Comparison-Outcome (PICO) framework,^12^ the estimand framework provides explicit guidance on how intercurrent events could be managed in the statistical analyses. Despite the potential of the estimand framework to improve the specification and interpretation of treatment effects, previous reviews consistently show that the uptake of the estimand framework in clinical trials has been slow.^13^^,^^14^ They similarly found that trials rarely specify the estimand of interest and, when specified, their descriptions are often incomplete or inaccurate^15^ with limited explanation of how intercurrent events will be handled.^14^ This ambiguity limits stakeholders’ ability to make well-informed decisions, as they are left to infer the intended treatment effect from reported results rather than having it explicitly stated.^16^

Anaesthesia trials are particularly susceptible to such ambiguity because intercurrent events are both common and intrinsic to perioperative care. Importantly, whether an event should be considered an intercurrent event, and which estimand strategy is most appropriate, depends on the scientific question, trial design, and intended interpretation of the treatment effect. Despite this, no structured guidance exists to help researchers prospectively identify and address these intercurrent events. Although a recent tutorial paper illustrated the framework’s application using a hypothetical anaesthesia trial (see Table 1), this remains conceptual.^9^ Reflecting these limitations, while the framework has been adopted in clinical trials in other fields,^17^, ^18^, ^19^, ^20^, ^21^ very few published examples currently exist in anaesthesia.^22^^,^^23^ In this paper, we addressed these critical gaps by (i) conducting a scoping review to evaluate the current use of the estimand framework in anaesthesia clinical trials and (ii) developing a practical framework to prospectively identify intercurrent events likely to occur in anaesthesia trials across different intervention-outcome settings.

**Table 1:** Example of an estimand and its attributes (adapted from the tutorial paper by De Silva and Colleagues^9^). This example is drawn from a tutorial paper illustrating the application of the estimand framework in anaesthesia research. The framework, introduced by the ICH E9(R1) addendum, aligns trial objectives with study design, conduct, analysis, and interpretation to clarify how intercurrent events such as treatment discontinuation, rescue medication use, or death affect the treatment effect being estimated. 3D-CAM, 3-dimension confusion assessment method; ICH, International Council for Harmonisation of Technical Requirements for Pharmaceuticals for Human Use.

Estimand attribute	Definition	Example
Population	The population addressed by the research question or the target population of interest	Males and females aged 60 yr or older scheduled for noncardiac surgery expected to last over 2 h, with a minimum of one planned overnight hospital stay
Treatment condition(s)	The treatment of interest and the comparator	Propofol versus sevoflurane for maintenance of anaesthesia
Outcome variable	The outcome variable measured for each patient to answer the research question of interest	Incidence of postoperative delirium, assessed twice daily with the 3D-CAM up to postoperative day 3 or discharge
Population level summary measure	The population-level summary measure used to compare the different treatment conditions	Absolute difference in the incidence of postoperative delirium between arms
Handling of intercurrent events	The strategies used to handle intercurrent events:	This trial included several intercurrent events such as the use of rescue medication, treatment switching, post-randomisation clinical events, and in-hospital death with each addressed using multiple strategies. For example, for the postoperative use of rescue medication (e.g. quetiapine for delirium), the distinct estimands are:
• Treatment policy strategy	The treatment effect is estimated regardless of whether the intercurrent event occurs.	What would happen if all patients were analysed as randomised, even if they used rescue medication? Appropriate when the trial aims to estimate the effect of assigning an intervention in routine clinical practice, reflecting real-world use where rescue medication is expected and part of usual care.
• Hypothetical strategy	The treatment effect is defined under a hypothetical scenario in which a specific intercurrent event is assumed not to occur, allowing the intervention effect to be interpreted independently of that event.	What would happen if, hypothetically, no patients used rescue medication? Appropriate when the research question asks what the treatment effect would be in a situation where the rescue medication was not used, and where it is realistic that rescue medication is not routine care.
• Composite strategy	The intercurrent event is incorporated into the outcome definition itself.	What would happen if rescue medication is considered part of the outcome? Appropriate when the use of rescue medication represents a clinically meaningful outcome or failure and should therefore be incorporated directly into the outcome definition instead of being analysed separately.
• Principal stratum strategy	The treatment effect is estimated only in those patients for whom the intercurrent event would not occur under either treatment.	What would happen if only a subpopulation of patients is considered as defined by their potential use of rescue medication? Appropriate when interest lies in understanding the treatment effect within a subpopulation defined by whether rescue medication would not be used under either treatment, acknowledging that this estimand targets a specific (often unobservable) patient subpopulation.
• While-on-treatment strategy	The treatment effect is defined for the period up to the occurrence of the intercurrent event.	What would happen if outcome until the use of rescue medication is considered? Appropriate when the research question focuses on the effect of treatment under the intended treatment pathway prior to the occurrence of rescue medication (i.e. while-not-on-rescue medication), and when outcome data collected after the start of rescue medication are considered less relevant to the estimand of interest. In this example, use of rescue medication, treatment switching, and post-randomisation clinical events are addressed using the treatment policy strategy and in-hospital death using the while-on-treatment (‘while alive’) strategy.
Corresponding research question (Primary estimand)	This trial aims to assess whether propofol is superior to sevoflurane in reducing postoperative delirium among patients aged 60 yr or older undergoing major noncardiac surgery lasting more than 2 h, with at least one planned postoperative night in hospital, regardless of rescue medication use, treatment switching, or post-randomisation clinical events, using twice-daily 3D-CAM assessments up to postoperative day 3 or death, whichever occurs first, and estimating the treatment effect as the difference in delirium risk between groups.	

## Methods

We conducted a scoping review following the Joanna Briggs Institute methodology for scoping reviews^24^ and reported our findings according to the Preferred Reporting Items for Systematic Reviews and Meta-Analyses extension for Scoping Reviews (PRISMA-ScR) checklist.^25^ The PRISMA-ScR checklist is available in Supplementary Table 1. In line with scoping review methodology, a risk-of-bias assessment was not performed, and no ethics approval was required. The protocol for this scoping review was prospectively registered on the Open Science Framework^26^ and is available in Supplementary File 1.

### Eligibility criteria

We included RCTs involving at least 100 human participants undergoing anaesthesia and surgery. We excluded phase 1 and 2 trials and studies involving minimally invasive or purely diagnostic gastrointestinal endoscopy. In addition, we excluded reviews, meta-analyses, sub-studies, editorials, letters, interim analyses, and preliminary studies, including pilot and feasibility studies. Where publicly available, study protocols and statistical analysis plans corresponding to the included RCTs were obtained. Full inclusion and exclusion criteria are provided in Supplementary Text 1.

### Information sources and search strategy

We searched for RCTs published between January 2020 and June 2024 in ten high-impact journals, including six general medical journals (Annals of Internal Medicine, Journal of the American Medical Association, New England Journal of Medicine, PLOS Medicine, The BMJ, and The Lancet) and four anaesthesia journals (Anaesthesia, Anaesthesia and Analgesia, Anaesthesiology and British Journal of Anaesthesia).

An initial limited search was conducted across MEDLINE, Embase and the Cochrane Central Register of Controlled Trials using ‘anaesthesia’, ‘randomised controlled trial’, and ‘estimand’ to identify keywords and index terms. We then developed two separate search strategies, tailored to medical and anaesthesia journals, combining keywords, Medical Subject Headings terms, and free text terms using Boolean operators, guided by the eligibility criteria. Full search strategies are provided in the Supplementary Text 2.

### Study selection

Two reviewers (GS, RG) independently screened titles, abstracts, and full texts against the eligibility criteria. Disagreements were resolved by a third reviewer (ADS). The list of included studies is provided in the Supplementary Table 2.

### Data extraction and analysis

Data were extracted by GAS into a REDCap database hosted at The University of Melbourne, which was piloted on 10 records.^27^^,^^28^ A random 50% sample was cross-checked (ADS) to validate the extracted data, and all trials were reviewed to validate intercurrent events and categorisations of interventions and outcomes (SL). Discrepancies were resolved by discussion with third reviewers (SB, KL) where necessary. From the eligible studies, data were extracted on trial characteristics, use of the term ‘estimand’, citation of the ICH E9(R1) addendum, attributes of the primary estimand, and occurrence of intercurrent events and strategies for handling them. We also recorded the availability of the protocol and statistical analysis plan in the public domain and whether estimands were specified within these documents. If estimands were not reported in these documents, we assessed each estimand attribute using information available in the main publication.

### Outcomes

After previously published reviews on estimands in clinical trials,^13^^,^^14^ we assessed whether each attribute of the primary estimand was explicitly stated, could be unambiguously inferred or was not inferable for each trial, based on information in the main trial publication. Where studies specified more than one primary endpoint, we selected the endpoint used to determine the sample size as the primary endpoint. We chose the primary estimand following a standardised decision framework. If the trial explicitly named a primary estimand, we used that. If no primary estimand was specified or more than one was named, we inferred the primary estimand from the description of the main analysis of the primary outcome. Where uncertainty remained, we reviewed the trial objective to clarify the targeted treatment effect and identify the primary estimand.

Attributes were considered as ‘stated’ when they were clearly described in the primary estimand or in the trial objective. For example, if the trial objective or the primary estimand explicitly specified the population as ‘adult patients (18 yr or older) undergoing laparoscopic or breast surgery who are eligible for randomisation’, we recorded the population attribute as ‘stated’. Attributes were deemed ‘inferable’ if explicit mention in the primary estimand or in the trial objective was lacking but could be unambiguously determined through information in the statistical analysis or elsewhere in the main trial publication. For example, if the population was not explicitly stated in the trial objective or the primary estimand, but the study was analysed using an intention-to-treat (ITT) principal^10^ (i.e. assess the effect in all randomised patients according to planned treatment), we inferred the population as ‘all eligible participants based on the ITT description of the analysis set’. Attributes were recorded as ‘not inferable’ when their definition could not be determined from any reported information. For example, when trials excluded participants who experienced treatment deviations, we recorded the population attribute as ‘not inferable’ because of the ambiguity about whether the target population corresponded to a hypothetical population (as if no deviations occurred) or a principal-stratum population (subpopulation of participants who would always adhere).

We also extracted information on intercurrent events that could be identified and strategies used to handle them. Following the ICH E9(R1) guidelines, we classified the handling strategies as treatment policy, hypothetical, composite, principal stratum and while-on-treatment, based on stated or inferred information (Table 1). Handling strategies were considered ‘stated’ if the strategy was explicitly stated, ‘inferable’ if we could infer the strategy from the available information, and ‘not inferable’ when no details were available.

Finally, we classified the overall estimand as ‘stated’ when all five attributes were clearly stated in the primary estimand description or the trial objective, ‘inferable’ when all five attributes were either stated or could be unambiguously inferred, or ‘not inferable’ if at least one of the attributes was not inferable. Where available, we recorded whether the study protocols and statistical analysis plans specified estimands.

### Framework development

Informed by the scoping review, we developed a framework to prospectively map likely intercurrent events in anaesthesia trials across different intervention-outcome settings. For each included study, we categorised interventions using Elliot and colleagues’^29^ typology, and primary outcomes using the Standardised Endpoints in Perioperative Medicine (StEP) domains^30^, ^31^, ^32^, ^33^, ^34^, ^35^, ^36^, ^37^, ^38^, ^39^ and identified the list of intercurrent events that occurred. We then determined the intervention-outcome combinations represented across all included studies and mapped each combination to the intercurrent events observed in studies investigating the same combination. This process allowed us to summarise which intercurrent events commonly arise for specific intervention and outcome types in anaesthesia research. These data were subsequently organised into an interactive web-based framework that enables users to select an intervention or outcome category and view the potential intercurrent events that are most frequently associated with that combination. The framework is designed as a practical tool to help researchers prospectively identify and anticipate intercurrent events when defining estimands or designing anaesthesia trials.

### Statistical methods

We summarised data descriptively using frequencies and percentages, and all analyses were performed using R version 4.4.0.^40^^,^^41^

## Results

We performed the initial search on August 16, 2024 and the final search on October 15, 2024, which retrieved 4670 records. After removing duplicates, 1684 unique records remained. Of these, 1545 were excluded during title, abstract and full-text screening based on predefined eligibility criteria. This led to 139 eligible trials being included in our review. Reasons for exclusion at full-text screening are detailed in the PRISMA flow diagram in the Supplementary Fig. 1.

Of the 139 included trials, 126 (91%) were published in anaesthesia journals and 13 (9%) in general medical journals. Eleven (8%) were funded by pharmaceutical companies, 128 (92%) were funded by other entities, such as academic institutions, government or public agencies, charitable foundations, and collaborative trial networks. The protocol was publicly available for 53 (38%) trials, available upon request for 20 (14%) trials, and not publicly available for 15 (11%) trials. Availability could not be verified in 51 (37%) trials. The statistical analysis plan was publicly available for 33 (24%) trials. Only 79 (57%) trials stated compliance with the Consolidated Standards of Reporting Trials (CONSORT) guidelines,^42^ and none referenced the estimand framework or the ICH E9(R1) addendum in their main trial publication, protocol, or statistical analysis plan. Further trial characteristics are described in the Supplementary Table 3.

### Primary estimand

**Fig 1:**
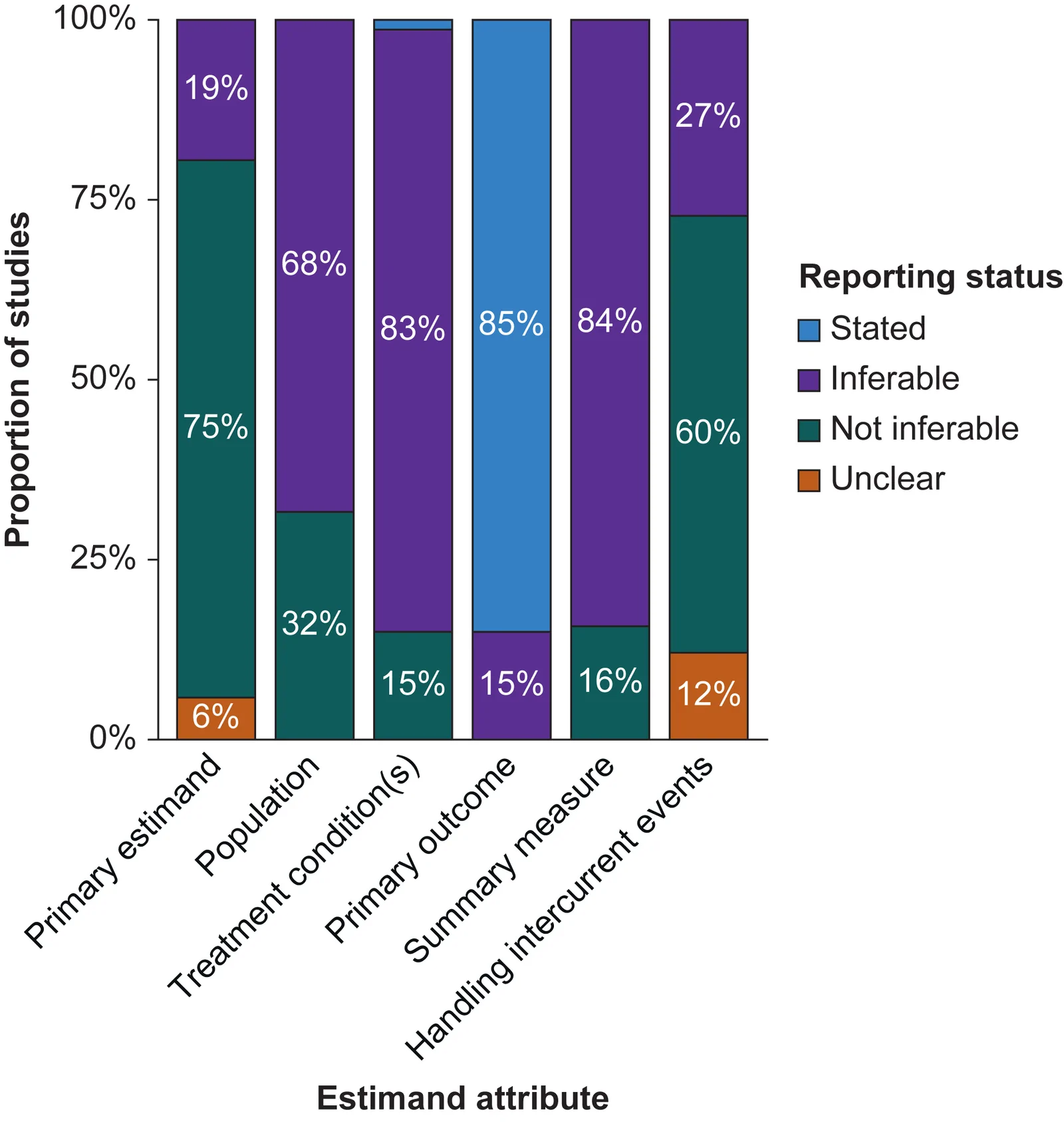
Reporting of primary estimand attributes across included trials.

None of the included trials explicitly stated the primary estimand, although it was inferable in 27 (19%), not inferable in 104 (75%) and unclear in eight (6%) trials, where reporting was ambiguous or it was uncertain whether any intercurrent events had occurred (Fig. 1). Among the five estimand attributes, the primary outcome was most often stated in the trial objective, whereas the treatment condition(s), population, summary measure, and intercurrent event handling strategies were rarely stated explicitly. The primary outcome could be determined in all trials, with 118 (85%) explicitly stating it in the trial objective. In contrast, the target population was not inferable in 44 (32%), the treatment condition(s) in 21 (15%), the summary measure in 22 (16%), and the handling of intercurrent events in 84 (60%) trials (Fig. 1).

### Intercurrent events

**Fig 2:**
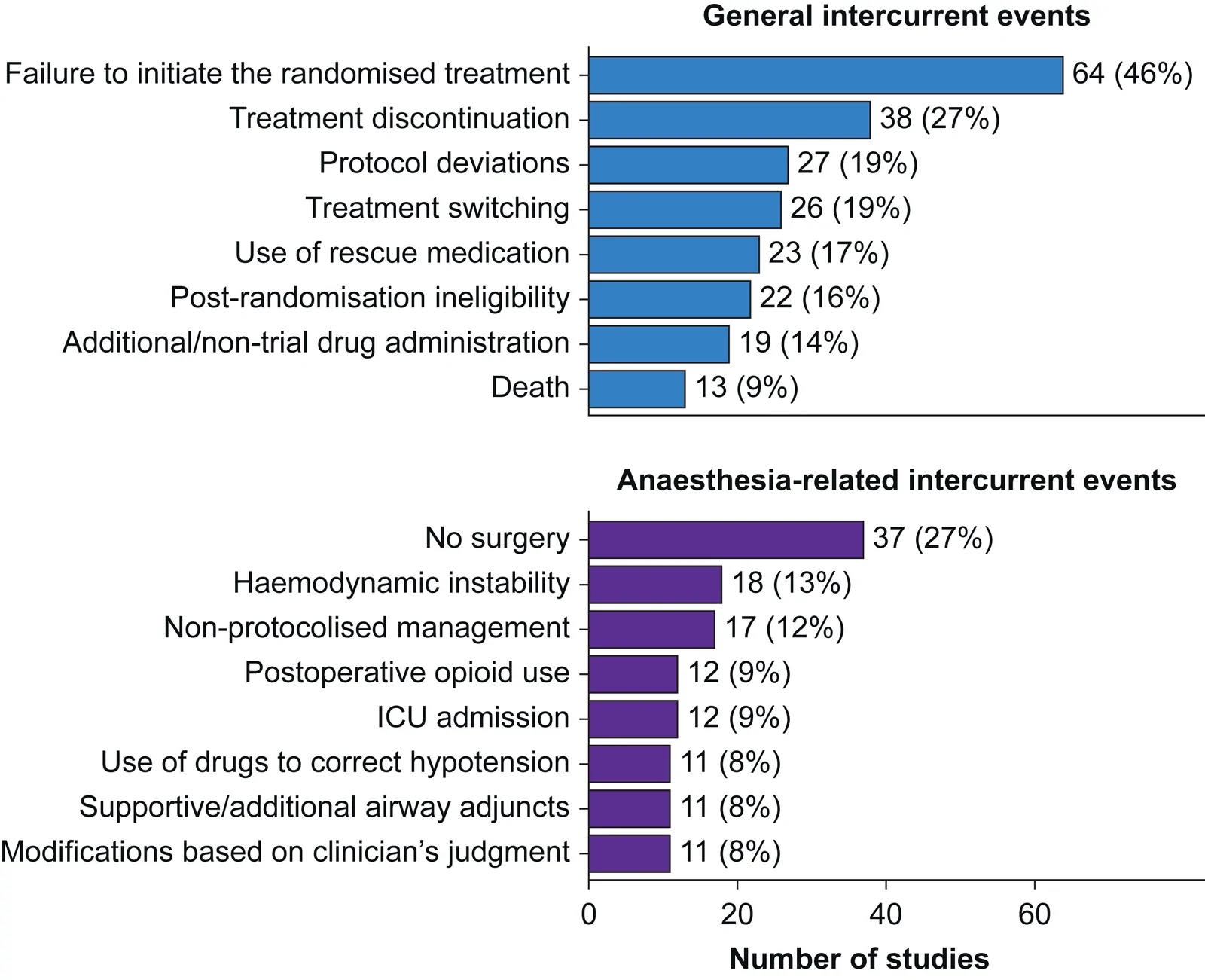
Most frequently reported intercurrent events in anaesthesia trials. The intercurrent events of ‘no surgery’ and ‘failure to initiate treatment’ may represent the same event (e.g. surgery cancellation resulting in failure of treatment initiation) or not. Some protocol deviations may constitute intercurrent events if they occur after randomisation and affect interpretation of the treatment effect. ‘Post-randomisation ineligibility’ captures instances where participants are discovered after randomisation to be ineligible for the trial (e.g. misapplied inclusion/exclusion criteria). Haemodynamic instability (e.g. hypotension or bradycardia) may function either as an intercurrent event or as a mediator.

None of the studies explicitly stated intercurrent events in the trial objective (Fig. 1). However, intercurrent events were identifiable based on other published information in 122 (88%) trials. In 17 (12%) trials, it remained unclear whether intercurrent events occurred or were relevant. The most frequent generic intercurrent events were: not receiving the randomised intervention (64 trials [46%]), treatment discontinuation (38 [27%]), protocol deviations (27 [19%]), treatment switching (26 [19%]), and use of rescue medication (23 [17%]) (Fig. 2). Detailed definitions of these intercurrent events, with proposed handling strategies, are presented in Table 2.^43^, ^44^, ^45^

**Table 2:** Common intercurrent events identified from the included studies that are applicable across clinical trials.

Intercurrent event	What is it?	Why is it an intercurrent event?	Considerations for handling strategy
Treatment discontinuation	Treatment discontinuation indicates the premature cessation of the assigned treatment. This may occur because of safety reasons, lack of efficacy, personal reasons or a range of other clinical reasons.	Classified as an intercurrent event because it happens after randomisation and changes the intended treatment pathway. This can disrupt the interpretation of the treatment effect, especially when it reflects tolerability.	When discontinuation is expected and frequent, applying the treatment policy strategy may be suitable. The hypothetical strategy can instead be used to estimate the treatment effect under the scenario in which patients had continued treatment, provided that this hypothetical scenario is plausible.
Protocol deviation	A protocol deviation refers to any divergence from, or non-adherence to, the study design or procedures as specified in the approved protocol.^43^ A few common examples include dosing and administration deviations, inclusion or exclusion criteria violations, procedure-related deviations, informed consent issues, randomisation issues, etc.	Protocol deviations may become an intercurrent event if they interfere with the intended treatment pathway.^44^ For example, when protocol allows discontinuation of treatment after an adverse response, it does not constitute a protocol deviation. Yet, this remains as an intercurrent event. However, a protocol deviation like rescheduling a study visit may not be an intercurrent event depending on its impact on the primary outcome.	Treatment policy strategy can be used to estimate the treatment effect regardless of protocol deviations. However, these protocol deviations usually result in missing data. In order to analyse missing data due to protocol deviations, options include defining whether the treatment effect of interest is under complete adherence or in real-world practice. Afterwards, patient data can be categorised into pre- and post-deviation events. Clear assumptions about post-deviation outcomes should then be specified, and statistical techniques like multiple imputation can be used to estimate the treatment effect.^45^
Treatment switching / Crossover	Treatment switching (or ‘crossover’) refers to when a participant leaves their assigned treatment for an alternative (e.g. from active to placebo or vice versa) during the trial. It can occur for various reasons, including efficacy concerns, safety concerns, or preference.	Classified as an intercurrent event as treatment switching occurs after randomisation and affects the interpretation of the treatment effect of interest. It can limit the ability to estimate the causal treatment effect.	The treatment policy strategy can be considered when the focus is to assess the treatment effect regardless of switching. Alternatively, the hypothetical strategy can be used when the goal is to evaluate the treatment effect had switching been avoided.
Use of rescue medication	Use of rescue medication refers to administering rescue therapy to manage uncontrolled symptoms or complications that the trial drug cannot sufficiently manage.	This is considered an intercurrent event as it represents a departure from the intended treatment plan and may affect the interpretation of the intended treatment effect by introducing additional therapeutic effects.	When the use of rescue medication is an outcome of interest, the composite strategy can be applied. Alternatively, the hypothetical strategy can be used to estimate the outcome in the absence of rescue therapy. If rescue medication is expected in routine care, the treatment policy strategy may be the most appropriate.

Several intercurrent events were common in the included anaesthesia trials. The most common were surgery cancellation (37 trials [27%]), haemodynamic instability (18 [13%]), non-protocolised anaesthesia management (17 [12%]), postoperative use of opioids as rescue medication (12 [9%]), planned or unplanned ICU admission (12 [9%]), use of supportive or additional airway adjuncts (11 [8%]), and modifications based on the clinician’s judgement (11 [8%]) (Fig. 2).

Strategies for intercurrent event handling could be inferred in 38 (27%) trials. The most commonly used strategy was the treatment policy strategy, where the analysis uses data after the intercurrent event, and thus the treatment effect is estimated regardless of the intercurrent event. The composite strategy was the next most commonly used strategy, where the intercurrent event (most commonly death) is directly incorporated into the primary endpoint.

### Framework for anaesthesia trials

Informed by these findings, we developed a framework to identify and categorise intercurrent events in anaesthesia trials, encompassing both generic (encountered across trials in diverse clinical contexts) and anaesthesia-related events. Using consistently classified interventions and outcomes, we provide a structured way to map intercurrent events to specific intervention-outcome combinations, forming the basis of the framework. Supplementary Fig. 2 illustrates how users can navigate the framework to explore intercurrent events across different intervention-outcome combinations. Accordingly, Table 3^46^, ^47^, ^48^, ^68^ begins with the intervention category and the corresponding outcome, followed by the intercurrent event and the rationale for why that event is considered an intercurrent event in this context. The final column provides illustrative examples of plausible estimand strategies for handling these events. To facilitate practical use of the framework, we also developed an interactive web-based application that allows users to explore intervention-outcome combinations and their associated intercurrent events in anaesthesia trials: anaesthesia-estimands.shinyapps.io/estimand_website/

**Table 3:** Examples of intercurrent events related to anaesthesia trials identified from the included studies. AKI, acute kidney injury; BIS, bispectral index; ETT, endotracheal tube; EMG, electromyography; PECS II, pectoral nerves; PONV, postoperative nausea and vomiting; QoR, quality of recovery; TIVA, total intravenous volatile anaesthesia. ∗These estimands are illustrative and may vary depending on the trial objectives, protocol, and clinical context.

Intervention	Outcome	Intercurrent event	Justification	Plausible estimands∗
**Depth of anaesthesia** ***Closed-loop BIS-guided anaesthesia***	Postoperative neurocognitive disorders^46^	***Failure to receive allocated intervention*** e.g. Surgery cancellation	Not receiving the allocated treatment (or having surgery cancelled) indicates a treatment initiation failure. This can result from worsening patient condition, physician or patient decision, or administration issues. It can be classified as an intercurrent event because it prevents the initiation of the intended treatment and changes the interpretation of the primary outcome.^47^	Principal stratum estimand – treatment effect in the subpopulation of patients whose surgery would not be cancelled under either treatment, commonly used when participants who do not initiate treatment due to surgery cancellations can be identified and when treatment initiation failures are unrelated to treatment assignment.^47^
**Administration of regional injection *PECS II block***	Multidimensional QoR score^48^			
**IV fluids** ***Goal-directed fluid therapy***	Major complications^49^			
**Supplemental oxygen** ***Oxygen and antioxidants***	The degree of myocardial injury^50^			
**TIVA**	Intraoperative blood glucose concentrations^51^	***Protocol deviations, non-protocolised management or rescue medication/treatment (depending on study protocol)*** e.g. Use of drugs to correct hypotension	Intraoperative hypotension is associated with stress hyperglycaemia. This may be mitigated by using vasopressors.	Treatment policy estimand – treatment effect regardless of the use of hypotension-correcting drugs, reflecting the effect of the anaesthetic regimen under routine clinical practice where such treatments may be administered.
***Remifentanil infusion***				
***Dexmedetomidine***	Incidence of AKI^52^		The use of vasopressors can reduce renal blood flow via vasoconstriction, potentially increasing the risk of AKI.	
***Opioid-Free anaesthesia protocol***	PONV^53^	***Common clinical events that may affect occurrence or measurement of the outcome*** e.g. Haemodynamic instability	Prolonged hypotension is independently associated with increased risk of PONV. In this study, events were measured and subsequently treated.	Treatment policy estimand – treatment effect regardless of haemodynamic instability and its management, reflecting the effect of the intervention under routine clinical practice where such events may occur.
***Dexmedetomidine***	Incidence of AKI^52^		Hypotension is associated with increased risk of AKI. In this study, hypotension was treated using vasopressors.	
***Dexmedetomidine, propofol vs sevoflurane*** ***General anaesthetic regimen***	Incidence of delirium^54^^,^^55^ Postoperative pulmonary complications^56^	***Common clinical events that may affect occurrence or measurement of the outcome*** e.g. Unplanned ICU admissions	Unplanned ICU admissions may complicate the assessment of postoperative delirium and are associated with postoperative pulmonary complications due to acute stress, pain, and respiratory compromise.	Composite estimand – treatment effect on a composite outcome, defined to include the occurrence of the intercurrent event (e.g. a composite of postoperative pulmonary complications or unplanned ICU admissions), with unplanned ICU admissions considered a clinically meaningful indicator of treatment failure.
**Videolaryngoscopy**	First attempt success rate^57^, ^58^, ^59^, ^60^ Incidence of insufficient signal electromyogram amplitude level^61^ Best glottic visualisation^62^	***Protocol deviations, non-protocolised management or rescue medication/treatment (depending on study protocol)*** e.g. Use of external manipulations	Patient positioning and external laryngeal manipulation can change glottic visualisation, and use of these manipulations can improve or impair successful intubation. Alterations to head positioning (e.g. flexion or extension) can affect the ETT-electrode placement, thus affecting laryngeal EMG signals.	Treatment policy estimand – treatment effect regardless of external manipulations such as patient repositioning or external laryngeal pressure, reflecting the performance of videolaryngoscopy under routine clinical practice where these manoeuvres are commonly used.
**Spinal anaesthesia with targeted sedation based on BIS values**	Incidence of delirium^63^	***Protocol deviations, non-protocolised management or rescue medication/treatment (depending on study protocol)*** e.g. Use of drugs that may promote/ameliorate delirium	Non-protocolised management may permit use of drugs associated with increased postoperative delirium such as benzodiazepines, steroids or opioids.	Treatment policy estimand – treatment effect regardless of non-protocolised medications that may influence delirium, reflecting the effect of the intended anaesthetic and sedation strategy under routine clinical practice where such medications may be used if clinically indicated.
**General anaesthesia**	Incidence of delirium^64^^,^^65^	***Common clinical events that may affect occurrence or measurement of the outcome*** e.g. Haemodynamic instability	Haemodynamic instability due to unrelated events such as bleeding can lead to reduced cerebral perfusion, increasing the risk of postoperative delirium. In this study, hypotension was managed with fluid and vasopressor administration, and adjustments to anaesthetic depth and epidural dose.	Hypothetical estimand – treatment effect in a hypothetical scenario in which haemodynamic instability does not occur, assuming that instability arising from unrelated events such as bleeding can be managed with fluids, vasopressors, or adjustments to anaesthetic depth, allowing the treatment effect to be estimated as if such instability were avoided.^44^
**Pre-procedural medications** ***Clonidine vs midazolam***	Postoperative negative behavioural changes^66^	***Common clinical events that may affect occurrence or measurement of the outcome*** e.g. Postoperative pain	Postoperative moderate to severe pain in children is strongly associated with general anxiety, regression-aggression, eating, and sleep-related behavioural changes.	Composite estimand – treatment effect on the composite outcome of having postoperative negative behavioural changes or postoperative pain, with postoperative pain considered a clinically meaningful indicator of treatment failure.
**Perioperative medications** ***Dexmedetomidine***	Postoperative negative behavioural changes^67^			
**Airway management** ***Mechanical ventilation with low tidal volume***	Postoperative pulmonary complications^68^	***Common clinical events that may affect occurrence or measurement of the outcome*** E.g., Prolonged intubation or noninvasive ventilation	Prolonged intubation or noninvasive ventilation can influence how pulmonary complications are reported and may increase the risk of hospital-acquired infection and ventilator-associated pulmonary complications.	Composite estimand – treatment effect on the composite outcome of postoperative pulmonary complications, prolonged intubation or noninvasive ventilation, with these intercurrent events considered clinically meaningful indicators of treatment failure.

An example of a generic event would be failure to initiate assigned treatment, which may occur because of the deterioration of the patient’s condition, logistical constraints, or clinical reassessment. This constitutes an intercurrent event because it prevents delivery of the allocated intervention. Depending on the research question, this could be handled using a treatment policy strategy (including all randomised patients to reflect the pragmatic effect), a hypothetical strategy (estimating effects had all patients initiated treatment as planned) or a principal-stratum strategy (estimating the effects within the subpopulation of participants who would initiate treatment regardless of the assigned treatment arm). A comprehensive list of common generic intercurrent events, their explanations, and proposed handling strategies is presented in Table 2.^43^, ^44^, ^45^

Beyond these, we identified intercurrent events related to anaesthesia trials that occur within particular intervention-outcome combinations. As an example, in a multicentre RCT, the effects of sugammadex *vs* neostigmine for reversal of rocuronium-induced neuromuscular block were compared in high-risk older adults having surgery under general anaesthesia.^69^ The primary endpoint was a five-point pulmonary outcome score measured on postoperative days 1, 3, and 7. In our framework, this example represents an intervention in the general anaesthesia domain (Intervention main category), specifically drugs given at emergence (Intervention subcategory 1) for reversal of neuromuscular blockade (Intervention subcategory 2), with the outcome classified under pulmonary complications according to StEP domains.^39^ Of note, this study was designed before the E9(R1) addendum was published.

Several intercurrent events occurred in this trial that could influence or complicate the interpretation of the pulmonary outcome score. For example, some participants remained intubated at the end of surgery. While intubated, they could not provide the primary outcome (which was a score that included subjective shortness of breath), leading investigators to omit these participants from the analysis set. Excluding them introduces (non-random) missingness and selection bias. Based on the ICH E9(R1) estimand framework, we propose alternative strategies to handle this intercurrent event. If the researchers aimed to examine the treatment effect under a hypothetical scenario where all patients successfully achieved extubation at the intended assessment time, a hypothetical strategy could be applied. Under this approach, we would analyse the primary outcome assuming all participants were extubated as scheduled, with missing outcomes imputed using baseline and perioperative characteristics. This would enable estimation of the treatment effect unaffected by extended intubation. Alternatively, if the researchers aimed to estimate the treatment effect among patients who would have been successfully extubated as scheduled regardless of the assigned intervention (‘always extubated’), a principal-stratum strategy could be applied. Although this would generate a clinically understandable comparison, its validity depends on many unverifiable assumptions about principal-stratum membership. A treatment policy strategy might alternatively be considered to estimate the treatment effect regardless of whether prolonged intubation occurred. However, this strategy requires outcome data to be available from all participants, including those who experienced the intercurrent event. As prolonged intubation prevents the collection of the primary outcome, it leads to missing data and precludes the use of a treatment policy strategy to estimate the treatment effect. Further events that may have occurred in this trial which could represent potential intercurrent events are summarised in Table 4.^69^ This table presents their corresponding descriptions and proposed handling strategies according to the estimand framework, from the perspectives of both patients and healthcare providers, and was adapted from the approach of Pham and colleagues^70^ for presenting intercurrent events in disease-specific trials.

**Table 4:** Example of intercurrent event handling in a multicentre randomised controlled trial.^69^ Ledowski and colleagues^69^ (2021) conducted a multicentre randomised controlled trial comparing sugammadex and neostigmine for reversal of rocuronium-induced neuromuscular block in high-risk older adults (ASA physical status III–IV) undergoing surgery under general anaesthesia. The primary endpoint was a five-point pulmonary outcome score assessed on postoperative days 1, 3, and 7. This table summarises events that may have occurred in this trial and could represent potential intercurrent events, together with proposed handling strategies aligned with the estimand framework. COPD, chronic obstructive pulmonary disease. ∗The suitability of these proposed estimands depends on the specific clinical context and the plausibility of the assumptions required to support them.

Intercurrent event	What is it?	Why is it an intercurrent event?	How has it been handled in the trial?	Potential estimands∗	
				Estimand 1	Estimand 2
Administration of additional or non-trial drug	Use of drugs outside trial interventions (e.g. atropine with neostigmine; antibiotics for post-op infection)	May influence pulmonary outcomes through physiological or treatment-related pathways unrelated to the randomised comparison	Atropine was protocol-mandated with neostigmine; antibiotics prescribed at clinicians’ discretion. Effects implicitly included in observed outcomes	Treatment policy – estimate treatment effect regardless of all subsequent care	Hypothetical – estimate treatment effect as if no additional drugs were needed
Treatment switching/ Crossover	One patient received both reversal agents	Alters post-randomisation exposure, deviating from the allocated reversal	Excluded from analysis	Treatment policy – estimate treatment effect regardless of treatment switching	Principal stratum – estimate treatment effect in the subpopulation of participants who would not switch under either treatment
Hypoxaemia (SpO_2_ <90%)	Post-extubation desaturation requiring supplemental oxygen or escalation of care beyond routine protocols	Represents a deviation from expected postoperative recovery and can both mediate downstream pulmonary outcomes and lead to missing outcome data or protocol deviations (e.g. ICU transfer)	All extubated patients routinely received 6 L min^−1^ O_2_; extra oxygen given as needed. Desaturation only reported descriptively	Treatment policy – estimate treatment effect regardless of hypoxaemia	Composite – estimate treatment effect integrating hypoxaemia into composite pulmonary outcome
Clinical events (e.g. pulmonary oedema, renal/hepatic impairment, sepsis, COPD/asthma exacerbation, pain-related atelectasis)	Serious postoperative complications affecting respiratory status or recovery	Occur after randomisation and can affect pulmonary outcomes, though not directly part of the 5-point score	Managed per standard care (antibiotics, oxygen, supportive therapy); not incorporated into the pulmonary score	Treatment policy – estimate treatment effect regardless of the occurrence of these events	Composite – estimate treatment effect integrating clinical events into composite pulmonary outcome
Unplanned ICU admission	Postoperative ICU transfer for monitoring or complications	Care in the ICU may involve respiratory support (e.g. noninvasive ventilation), which can affect the incidence of pulmonary complications.	Two unexpected ICU admissions (sugammadex group); treated as observed events	Treatment policy – estimate treatment effect regardless of ICU admissions	Hypothetical – estimate treatment effect had ICU admission not occurred

See the Supplementary File 2 for the full framework.

## Discussion

Our review covering the 5 yr since the release of the ICH E9(R1) addendum reveals that adoption of the estimand framework is lacking in anaesthesia trials. The estimand framework was not reported in any of the 139 included anaesthesia trials published in high-impact medical and anaesthesia journals, and reporting of individual estimand attributes was often incomplete or missing.

Our finding is consistent with previous research in other contexts.^13^, ^14^, ^15^ In line with Kahan and colleagues,^13^ we found that trial objectives often describe the primary endpoint but omit the other four estimand attributes, particularly the intercurrent events. In contrast to Clark and colleagues’^15^ findings, however, where protocols increasingly included estimands following the ICH E9(R1) addendum, we found no evidence of estimand framework adoption in either published protocols or statistical analysis plans in anaesthesia trials published after 2019.

None of the trials explicitly stated their intercurrent event handling strategies. In 60% of trials, the handling strategy was not inferable, primarily because of participant exclusions from the analysis without indicating whether a hypothetical or principal-stratum strategy was intended. Unclear handling strategies were a major contributor to the ambiguity in research questions observed in 75% of trials, aligning with findings from earlier reviews.^14^ Where strategies were inferable, most trials implemented the treatment policy strategy overlooking intercurrent events, and others applied the composite strategy incorporating such events into the primary outcome. We could not infer the application of the other handling strategies based on what was reported in these papers.

The perioperative period is highly dynamic, with surgeons and anaesthetists making multiple, rapid decisions about a myriad of issues, including whether to proceed, what surgical approach to take, the type of anaesthesia required, and the response to intraoperative instability. Because of this, most anaesthesia trials do not highly protocolise management and allow clinician discretion, leading to many potential intercurrent events. Well-designed trials should consider these events *a priori*; determine whether these events are intercurrent events or mediators (i.e. post-treatment events that lie on the causal pathway between the intervention and the outcome); collect data about them; or protocolise key elements of care.

Through our review, we identified common intercurrent events applicable across clinical trials more broadly, such as treatment switching, treatment discontinuation, and use of rescue medications. In addition, we identified intercurrent events related to anaesthesia trials, including surgery cancellations, haemodynamic instability influencing the trial outcomes, non-protocolised anaesthesia management, and ICU admissions, all with markedly different handling approaches. For example, surgery cancellations were not treated as an intercurrent event in any of these trials and were managed in different ways.^47^ Whether such events are considered intercurrent events, and how they are handled, depends on the intended trial design and estimand. Similar events may be treated differently in explanatory *vs* pragmatic trials, depending on the clinical question of interest.^44^ Some trials excluded participants whose surgeries were cancelled, while others included them through an ITT analysis. Although widely used, both approaches address different clinical questions. Moreover, analyses performed on a subset of randomised participants based on post-randomisation exclusions such as non-adherence or protocol deviations makes them vulnerable to selection bias and may lead to patient information loss.^71^ Through the estimand framework, intercurrent events are defined alongside explicit handling strategies, allowing the research question to be specified precisely and aligned with stakeholder needs.

The strengths of our review include that we followed a pre-registered protocol using standardised, piloted data extraction forms.^26^ To ensure accuracy, a clinical expert (SL) independently verified the intercurrent events and the categorisation of interventions and outcomes across all trials. To our knowledge, this represents the first scoping review investigating estimand use in anaesthesia trials, providing novel insights into discipline-specific challenges. In addition, we developed a practical framework identifying anaesthesia-specific intercurrent events across different intervention-outcome combinations, contributing guidance for future anaesthesia trial planning.

Limitations include that we restricted our review to 10 high-impact journals, which could have overestimated reporting quality standards compared with broader journal coverage. Our findings were largely drawn from investigator-initiated trials, reflecting the uptake of the estimand framework in academic research as opposed to pharmaceutical industry research. Inferring estimand attributes was challenging because no trials explicitly stated them, demanding subjective interpretation of paper contents. However, this limitation was mitigated through a dual-reviewer process, with intercurrent events independently reviewed in all trials and other estimand attributes in a random 50% sample. Furthermore, some intervention and outcome categorisation involved subjective decision-making for items not aligning with established categories, potentially compromising reproducibility. Finally, our 2020–4 timeframe may not have captured ongoing longer-term trials that are yet to be published or ongoing trials that were designed after the ICH E9(R1) addendum. It should also be acknowledged that many of the trials included were likely designed prior to the release of the ICH E9(R1) addendum, which may partly explain the limited uptake observed.

The lack of explicit estimand use, even among trials published in leading journals, underscores the need for a cultural transformation in reporting. Notably, the recent CONSORT and SPIRIT 2025 update does not require an explicit statement of the estimand.^42^^,^^72^, ^73^, ^74^ Rather, it encourages reporting when trials have been designed using the estimand framework, whereas accepting that retrospective application to existing trials is challenging. To provide clarity in the treatment effect under investigation, anaesthesia trials must start adopting greater transparency through explicit specification of all estimand attributes in all trial publications. This also extends to improving protocol accessibility. In our sample, only 38% of trials had a publicly available protocol, limiting the ability to assess prespecified objectives, intercurrent event handling, and alignment between the planned and executed analyses. Clear guidance and dedicated training are essential for anaesthesia researchers to integrate estimands into trial design, ensuring research questions remain accessible and relevant to stakeholders.^9^^,^^44^^,^^75^, ^76^, ^77^, ^78^, ^79^, ^80^, ^81^ In some contexts, different stakeholder perspectives may necessitate multiple estimands.^10^^,^^82^, ^83^, ^84^ The choice of estimands should be informed by collaboration among trialists, clinicians, statisticians, and patients, rather than being treated as a purely statistical exercise. Mandating estimand specification across reporting guidelines, funders, and journals could help achieve greater consistency and clarity in trial findings. Together these strategies will lead to more robust evidence and will help clinicians, researchers and policymakers to make better decisions about patient care.

In conclusion, this study addresses critical gaps in anaesthesia trial methodology through two key contributions: a scoping review documenting the current lack of estimand framework implementation in anaesthesia trials, and a practical framework for prospectively identifying intercurrent events across different intervention-outcome settings. Together, these contributions offer anaesthesia researchers concrete steps toward designing more robust trials that generate reliable, clinically meaningful evidence.

## Authors' contributions

Conceptualisation, data curation, formal analysis, investigation, methodology, project administration, visualisation, writing - original draft: GAS

Validation, writing- reviewing and editing: SL

Investigation, validation, writing - reviewing and editing: RKG

Conceptualisation, methodology, supervision, writing - reviewing and editing: JAS, SB

Methodology, supervision, writing - reviewing and editing: KL

Conceptualisation, methodology, supervision, validation, writing - reviewing and editing: ADS

## Funding

National Health and Medical Research Council (NHMRC) Investigator Grant Leadership Level 1 (ID 1196068) to JAS, NHMRC Centre of Research Excellence grant (ID 1171422;, Australian Trials Methodology (AusTriM) Research Network) and a University of Melbourne Research Scholarship to GAS.

## Declaration of interests

KL was an author of studies included in this review. KL is an editor of the *British Journal of Anaesthesia*.
