# Supplementary material for: Defining estimands in anaesthesia clinical trials: a scoping review and framework
Source: Br J Anaesth. 2026 Apr 20;137(2):610–24. doi: 10.1016/j.bja.2026.03.026 (PMC13494091; doi:10.1016/j.bja.2026.03.026)
Supplement: Supplementary file 1 [file mmc1.docx]

### Supplementary File S1. Study protocol (registered on OSF)

OSF registration link - <https://osf.io/4njsr/overview>

**Estimands in anaesthesia clinical trials - The protocol for a scoping review**

### **Type of review ***

This can be, for example, a meta-analysis, evidence map, or a qualitative review. Also indicate whether you used any guidelines, tools, or checklists to prepare your protocol and, if so, which ones. For more information, see: Tricco, A. C., Tetzlaff, J., Moher, D. (2011). The art and science of knowledge synthesis. https://doi.org/frdpd2.

This is a scoping review protocol for the application of the estimand framework in anaesthesia clinical trials. This scoping review protocol was prepared in accordance with PRISMA-P guidelines and the PRISMA extension for scoping reviews ^1^.

### **Review stages ***

Indicate the stages in which you will conduct this review. Common stages are, in this order, the sections of this form: Search, Screening, Extraction, Synthesis. Sometimes other stages are distinguished, such as Preparation, Critical Appraisal, and Reporting. Additionally, it can be beneficial to include pilot stages for screening and extraction, while mentioning any updates to the preregistration. The stages could then look like: Preparation, Search, Pilot Screening (100 hits), Prereg Update, Screening, Pilot Extraction (10 sources), Prereg update, Extraction, Synthesis.

Preparation, Search, Pilot Screening (20 sources), Screening, Pilot Extraction (20 sources), Pre-registering, Extraction, Synthesis.

### **Current review stage ***

Indicate which stage from the “Review stages” item you are at this moment (i.e., when you freeze this registration). Note that in many contexts, only registrations in earlier stages are considered preregistrations. For example, you can indicate whether you started and/or finished with a certain stage as is customary for PROSPERO registrations. In addition, if this is not the first preregistration (but a second or third update, e.g., after pilot screening or pilot extraction), you can make that explicit here.

Extraction

### **Start date ***

Indicate the planned start date, or if you already started, the actual start date.

19/08/2024

### **End date ***

Indicate the planned end date, or if you already completed the review, the actual end date. You can use resources such as PredicTER.org to estimate how long a review will take to complete.

31/06/2025

### **Background ***

Introduce the topic of your review, its aims, and/or provide a short summary of known literature and what your review adds to this literature. You can describe why the review is needed, as well as which reviews already exist on this or related topics.

In recent years, there has been a significant increase in both investment and clinical trial activity in anaesthesia research globally ^2^. Within many of these anaesthesia clinical trials, numerous events occur after randomisation, such as the need for rescue medication or alternative treatments during the trial, participants switching treatments after assignment, or death at the hospital before outcome measurements are collected ^3^. These events, which are referred to as intercurrent events, can potentially influence study outcomes ^4^ and, consequently, trial findings. Further, it is often unclear how these intercurrent events are managed within trials, complicating the interpretation of results ^3^.

This is where the concept of estimands becomes invaluable. Introduced by the ICH E9 (R1) Addendum in 2019^5^, the estimand framework clearly defines the treatment effect. Estimands ensure that what is being estimated is relevant and aligns the study design and conduct, data collection, statistical analysis and interpretation of results with the trial objectives. By precisely defining the treatment effect of interest, the estimand framework ensures that all necessary data are collected in advance.

Given the importance of estimands in clinical trials, this scoping review aims to identify how the estimand framework is utilised in anaesthesia clinical trials published in major medical and anaesthesia journals.

### **Primary research question(s) ***

List the specific questions this review is meant to answer (i.e., the questions that ultimately informed the decisions made when designing the search strategy, and screening, extraction, and synthesis plans). You may find it helpful to refer to frameworks such as PICOS where appropriate to pinpoint your research questions. Note that all analyses pertaining to primary research questions should normally be reported in the final report.

To identify how the estimand framework is utilised in anaesthesia clinical trials published in major medical and anaesthesia journals.

### **Secondary research question(s) ***

List additional research questions that you will examine, but that took less central roles in informing the review’s design. Note that all analyses pertaining to secondary research questions should normally be reported in the final report.

1) How often the estimand framework has been explicitly stated

2) How well the attributes of the estimands are described

3) What types of intercurrent events are anticipated or were observed

4) How the intercurrent events were handled

### **Expectations / hypotheses ***

Describe any hypotheses (common for quantitative approaches) and/or expectations you have. These can pertain to your research questions, the types of sources you will find, social and political contexts, and contextual information that you know may color your interpretations and decisions (common for qualitative approaches).

We expect to have around 50% anaesthesia clinical trials in which the primary estimand is stated or inferable. This expectation is based on a previous scoping review conducted in a broader clinical context where they showed that out of 255 eligible randomised trials, the primary estimand could be determined in 46% of the trials ^6^.

### **Dependent variable(s) / outcome(s) / main variables ***

List the dependent / outcome / main variables you are interested in. If this review concerns one or more associations, list the outcome variable(s) or dependent variables. If this review does not concern one or more associations (e.g., in reviews of single variables such as prevalences, or descriptive reviews), list the main variables of interest here.

Not applicable.

### **Independent variable(s) / intervention(s) / treatment(s) ***

If this review’s research question(s) concerns one or more associations or effects, list the variable(s) that theoretically cause them or are assumed to otherwise explain the dependent variable(s) / outcome(s). If this is a manipulation, treatment, or intervention, make sure to describe it in full: that means also describing all groups, including any control group(s) or comparator(s).

The review will include all eligible randomised trials.

### **Additional variable(s) / covariate(s) ***

Here, list any additional variables you are interested in that were not included in the two lists above, such as covariates, moderators, or mediators.

Not applicable.

### **Software ***

List the software you will use for the review, for instance to store and screen search results, extract data, keep track of decisions, and to synthesize the results. Include version numbers and operating systems, if applicable.

Endnote 21 for storing the results

Covidence for screening

REDCap for data extraction

R for statistical analysis

### **Funding ***

List the funding sources for everybody that is involved in this review at this stage. If the work is unfunded, please state this as such.

This research is funded by a Melbourne Research Scholarship awarded to Gethmini Siriwardena.

### **Conflicts of interest ***

List any potential conflicts of interest (e.g., if there is a potential outcome of this review that can in any way have negative or positive effects for anybody involved in this review in terms of funding, prestige, or opportunities). If there are no conflicts of interest, please state this as such.

None

### **Overlapping authorships ***

Declare whether you expect that anyone involved in this review is a co-author of one of the studies that will likely be included in the review (based on your search strategy) and, if so, how you will address potential bias (i.e., that reviewer is not involved in screening, data extraction, quality assessment, or synthesis of that study). If you are confident that this does not represent a conflict of interest, explain why you think so.

Kate Leslie will be working as a third reviewer to resolve any conflicts in the screening and data extraction processes. Kate has co-authored the following study included in this scoping review.

Corcoran, T. B., et al. (2021). "Dexamethasone and Surgical-Site Infection." New England journal of medicine 384(18): 1731.

## Search Strategy

In this section, you register your search strategy: the procedures you designed to obtain all (potentially) relevant sources to review (e.g., articles, books, preprints, reports, case law, policy papers, archived documents).

### **Databases ***

List the databases you will search (e.g., ArXiv, PubMed, Scopus, Web of Science, PsycINFO, AGRIS, BioOne, PubChem). Note that these are different from interfaces (see next question).

PubMed (Medline)

Embase

Cochrane Central Register of Controlled Trials

### **Interfaces ***

For each database, list the interface you used to search that database (e.g., Ovid or EBSCO). Some databases are provided by the same organisation, in which case the interface can have the same name (e.g., PubMed, ArXiv). For more information about the distinction.

All databases were searched through Ovid.

### **Grey literature ***

List your strategies for locating grey literature (i.e., sources not indexed in the databases you search) such as pre-prints (e.g., disciplinary repositories such as ArXiv or PsyArXiv or university repositories using for example, DSpace), dissertations and theses, conference proceedings and abstracts, government/industry reports etc.

This review will focus only on published clinical trials and their protocols and statistical analysis plans. Therefore, we are not including grey literature.

### **Inclusion and exclusion criteria ***

List the specific inclusion and exclusion criteria that you used to inform your search strategy. Also list the framework(s) you used to establish your exclusion and inclusion criteria and use them to develop your search query, if any. Examples of the latter are PICO (Population, Intervention, Comparison, Outcome) and SPIDER (Sample, Phenomenon of Interest, Design, Evaluation, Research type), but many more exist.

Inclusion Criteria

- Published in English Language
- Published between 1/1/2020 - 30/6/2024
- Randomised controlled trials
- Human participants
- Phase 3 or 4 clinical trials
- Clinical trials involving patients who received anaesthesia prior to surgery
- Sample size greater than 100 patients
- Superiority trials, non-inferiority trials and equivalence trials
- Parallel group, crossover, factorial, individually randomised and cluster randomised designs
- Primary trial results published in:
  - Annals of Internal Medicine
  - British Medical Journal (BMJ)
  - Journal of the American Medical Association (JAMA)
  - The Lancet
  - New England Journal of Medicine (NEJM)
  - PLOS Medicine
  - British Journal of Anaesthesia
  - Anaesthesia
  - Anesthesiology
  - Anesthesia and Analgesia

Exclusion Criteria

- Published before or after the inclusion period
- Published in any language other than English
- Non-randomised trials
- Studies focused on pain medicine for post-operative pain management or chronic pain
- Clinical trials involving gastrointestinal procedures that are diagnostic or minimally invasive
- Sample size less than 100 patients
- Pilot studies and feasibility studies
- Reviews, meta-analyses, sub studies, editorials, or letters
- Interim analyses
- Trials in animals
- Phase 1 and 2 trials

### **Query strings ***

For each database/interface combination, list the query you will input (note that the available fields and operators can differ by database and by interface). The query string can be based on, for example, your inclusion criteria, the entities you want to extract (see “extraction”) and design requirements (e.g., qualitative studies, RCTs, or prevalence studies).

**OVID – EMBASE and OVID – Medline**

*Medical Journals*

Clinical trial filters including controlled, randomized, Phase 3 and Phase 4 trials

Anaesthesia-related subject headings and keywords

Analgesia-related terms

Terms related to artificial or assisted ventilation and airway management

Use of the term "estimand" across titles and abstracts

Filter to include a specific high-impact medical journal by title or ISSN

Limited the search from 2020 to 2024

*Anaesthesia journals*

Clinical trial filters including controlled, randomized, Phase 3 and Phase 4 trials

Filter to include a specific high-impact anaesthesia journal by title or ISSN

Limited the search from 2020 to 2024

**Cochrane Central Register of Controlled Trials**

*Medical Journals*

Anaesthesia-related subject headings and keywords

Analgesia-related terms

Terms related to artificial or assisted ventilation and airway management

Use of the term "estimand" across titles and abstracts

Filter to include a specific high-impact medical journals by title or ISSN

Limited the search from 2020 to 2024

*Anaesthesia Journals*

Filter to include a specific high-impact anaesthesia journal by title or ISSN

Limited the search from 2020 to 2024

### **Search validation procedure ***

Explain whether you plan to employ a search validation procedure, and if so, describe the procedure. For example, you can use a number of sources that you know your search strategy will have to turn up to validate your strategy and make adjustments if needed.

We validated our search strategy by checking with an industry expert to ensure that key anaesthesia journals published during the period were captured.

### **Other search strategies ***

List any additional search strategies you aim to employ, such as using the ascendancy approach (look through other sources cited in your included sources), the descendancy approach (look through the sources that cite your included sources using systems such as Crossref), or using other systems such as CoCites.

None

### **Procedures to contact authors ***

Describe your procedures for contacting authors. Will you contact authors? When? How will you follow-up on your first contact? Do you plan to share meta-data about those communications, and if so, how do you ask authors’ permission for that? Will you Note that templates are available at https://osf.io/q8stz/

Not applicable.

### **Results of contacting authors ***

Describe whether you plan to report the outcomes of contacting the authors (e.g., how many authors responded, how many authors sent data), and if so, how.

Not applicable.

### **Search expiration and repetition ***

Depending on how quickly the literature in an area expands, searches can have limited expiration dates; and for living reviews, repetition is planned regardless of ideas about expiration. Will you repeat your search (for example, in the case of a living review), and if so, how many months or years after your first search?

A search repetition will be done if it is older than 18 months.

### **Search strategy justification ***

Search strategies are often compromises, balancing pragmatic considerations with scientific rigour. Here, describe the justifications for your decisions about the databases, interfaces, grey literature strategies, query strings, author contact procedures, and search expiration date.

The databases chosen (Medline, Embase, and CENTRAL) ensure a vast coverage of literature, with Medline offering an extensive repository of peer-reviewed clinical studies, Embase providing robust indexing of European and international research, and CENTRAL focusing specifically on randomized controlled trials.

All searches were conducted using the OVID interface, selected for its advanced search capabilities and user-friendly design.

We have limited our search to higher impact factor journals.

Grey literature was not included to maintain a focus on peer-reviewed and indexed studies, ensuring consistency and quality in the data retrieved.

The query strings used were highly detailed, incorporating controlled vocabulary such as MeSH terms and free-text keywords, to capture a wide range of relevant studies while minimizing irrelevant results.

### **Miscellaneous search strategy details ***

Here, you can describe any details that are not captured in the other fields in this section.

This review will focus only on published clinical trials and their protocols and statistical analysis plans. A three-step search strategy will be used: 1) An initial limited search in PubMed was conducted to identify articles and keywords on the topic. The initial search strategy was developed using search terms that were obtained from similar studies done in the same field**.** A senior liaison librarian at the University of Melbourne (Australia) was consulted to develop the final search strategy; 2) The developed final search strategy will be conducted in EMBASE, Medline and COCHRANE databases using keywords and index terms. Two search systems will be used across the three databases for medical journals and anaesthesia journals separately.

### **Screening**

In this section, you register your screening procedure: the procedure you designed to eliminate all irrelevant sources from the results of the search strategy (and retain the relevant sources).

### **Screening stages ***

Describe the stages you will use for screening. For example, if you expect many hits, you may want to first screen based on titles only, in a second round also include abstracts and keywords, and in a third round screen based on full texts. Also indicate for each round whether the screening is done by a computer (e.g., AI), a human, or a computer supervised by a human. Don’t forget to describe the deduplication procedure, if you implement it.

Following the search, all identified citations will be collated and uploaded into a reference management and citation platform (Endnote) and duplicates will be removed. The references will then be imported into Covidence. The review will commence with a pilot phase where two authors will independently assess 20 abstracts to ensure the consistency of the inclusion and exclusion criteria. Titles and abstracts of all sources will then be screened by the two reviewers and assessed against the eligibility criteria. The full text of all the resulting citations will be assessed in detail against the inclusion criteria by the first reviewer and 20% of the full text will be screened by the second reviewer. Any disagreements that arise between the reviewers at each stage of the selection process will be resolved through discussion with a third reviewer.

### **Screened fields / blinding ***

Describe which bibliographic fields (e.g., title, abstract, authors) are visible during the screening, and which fields are blinded. For example, journal names, authors, and publication years can be hidden from screeners in an effort to minimize bias.

The title, abstract, journal name, publication years and authors are visible during the initial screening stage.

### **Used exclusion criteria ***

List the specific exclusion criteria that you apply in your screening to eliminate sources from the set of sources identified in your search. Note that inclusion criteria are typically used to inform the search strategy; during screening, as soon as an exclusion criterion is met, an entry is excluded, and so, inclusion criteria are reformulated into exclusion criteria where applicable.

Exclusion Criteria

- Published before or after the inclusion period
- Non-randomised trials
- Studies focused on pain medicine for post-operative pain management or chronic pain
- Clinical trials involving gastrointestinal procedures that are diagnostic or minimally invasive
- Sample size less than 100 patients
- Pilot studies and feasibility studies
- Reviews, meta-analyses, editorials, or letters
- Interim analyses
- Trials in animals
- Phase 1 and 2 trials

### **Screener instructions ***

List the instructions provided to the screener(s). You can also specify which file in the associated OSF project contains these instructions.

**Title and Abstract Screening**

1. Language: Is the article published in English?

- Yes: Keep screening.
- No: Stop screening.

2. Publication Date: Is the study published between 1/1/2020 and 31/6/2024?

- Yes: Keep screening.
- No: Stop screening.

3. Study Type: Is the study a randomised controlled trial?

- Yes: Keep screening.
- No: Stop screening.

4. Trial Phase: Is the study a Phase 3 or Phase 4 clinical trial?

- Yes: Keep screening.
- No: Stop screening.

5. Population: Does the study involve human patients who received anesthesia prior to surgery?

- Yes: Keep screening.
- No: Stop screening.

6. Sample Size: Is the sample size greater than 100?

- Yes: Keep screening.
- No: Stop screening.

7. Trial Design: Is the study design one of the following: parallel group, crossover, factorial, individually randomised, or cluster randomised?

- Yes: Keep screening.
- No: Stop screening.

8. Primary Trial Results Published In: Is the article published in one of the following journals:

1. Annals of Internal Medicine
2. British Medical Journal (BMJ)
3. Journal of the American Medical Association (JAMA)
4. The Lancet
5. New England Journal of Medicine (NEJM)
6. PLOS Medicine
7. British Journal of Anaesthesia
8. Anaesthesia
9. Anesthesiology
10. Anesthesia and Analgesia

- Yes: Keep screening.
- No: Stop screening.

**Final Decision:**

- **Include**: if all questions are answered "Yes."
- **Exclude**: If at least one answer is "No."

### **Screening reliability ***

For each screening round, list the number of screeners and the procedure used to ensure independent screening. This can also mean that you declare that you only use one screener, use multiple screeners that work together, or that you will not implement procedures to ensure that the screening is conducted independently. Also explain the test you will use, if any, to assess screener agreement.

Titles and abstracts will be screened by the two reviewers and assessed against the eligibility criteria.

The full text of the resulting citations will be assessed in detail against the inclusion criteria by the first reviewer and 20% of the full text will be screened by the second reviewer.

### **Screening reconciliation procedure ***

If you use more than one screener, describe the procedure to deal with divergent screener decisions for each screener round (e.g., through discussion or input from an additional screener).

Any disagreements that arise between the reviewers at each stage of the selection process will be resolved through discussion with a third reviewer.

### **Sampling and sample size ***

Describe whether you plan to use all sources included through the screening procedure, or whether you plan to sample from these sources (note that in most cases, all studies identified at this stage are kept). In case of the latter, describe the procedure you plan to use, the sample size analyses you conducted or will conduct, and the resulting required sample size if that is already available. If you plan to refrain from drawing conclusions, or draw more nuanced conclusions, describe that here as well. Finally, describe what you will do if a minimum required sample size or power is not reached (for your main analysis and any supplementary analyses).

All manuscripts meeting our eligibility criteria will be included.

### **Screening procedure justification ***

Screening procedures are often compromises, balancing pragmatic considerations with scientific rigour. Here, describe the justifications for your decisions about the screening rounds, blinding, in/exclusion criteria, assurance, and reconciliation procedures.

The titles and abstracts of all sources will be screened by two reviewers and any discrepancies and uncertainties that arise will be resolved by discussions with another third reviewer.

It will be guided by the inclusion and exclusion criteria.

### **Data management and sharing ***

Describe whether and how you plan to share the sources you obtained from the searches in the databases (see Search Strategy) and the decisions each screener made in each screening round. List both the file format (e.g., BibTeX, RIS, CSV, XLSX), the repository, and any potential embargos or conditions for access.

Data will be managed in Covidence. The results will be made available in .csv format upon reasonable request.

### **Miscellaneous screening details ***

Here, you can describe any details that are not captured in the other fields in this section.

Not applicable.

## Extraction

In this section, you register your plans for data extraction: the procedures you designed to extract the data you are interested in from the included sources. Examples of such data are text fragments, effect sizes, study design characteristics, year of publication, characteristics of measurement instruments, final verdicts and associated penalties in a legal system, company turnovers, sample sizes, or prevalences.

### **Entities to extract ***

List all entities that will be extracted from each included source. Entities can be, for example, 1) variables such as values of independent and dependent variables, and potential moderators (e.g., means, standard deviations); 2) estimations of associations between variables or effect sizes (e.g., Pearson’s r or Cohen’s d); 3) qualitative data fragments (e.g., interview material or synthesized themes); 4) descriptions of the used methods such as the included studies’ designs, sample sizes, sample characteristics, time between data collection sessions, and blinding procedures; 5) metadata such as authors, institutions, and year of publication; 6) and (other) risk of bias indicators.

Data will be extracted on the background of the study and the intercurrent events.

### **Extraction stages ***

Describe the stages you will use for extraction. Examples of stages are: a training stage, a reliability verification stage, and a final extraction stage; or first extracting primary data and in a second stage risk of bias information; or two extractors working sequentially or in parallel. Also indicate for each stage whether the extraction is done by a computer (e.g., AI), a human, or a computer supervised by a human.

Training stage - A data extraction form was developed in REDCap and tested by 2 authors using a sample of 20 articles ^7, 8^.

Final extraction stage – A double data extraction will be conducted, and all the data extraction will be done using REDCap.

### **Extractor instructions ***

List the instructions provided to the extractors (i.e., those performing the data extraction). You can also specify which file in the associated OSF project contains these instructions.

You may attach up to 5 file(s) to this question. Files cannot total over 5GB in size.

### **Extractor masking ***

If masking is used, describe the procedure used to blind extractors from the research questions, hypotheses, and/or specific roles of each entity to extract in this review. For example, extractors can be research assistants who are not informed of the study’s background or research questions, but who are trained to extract entities using the coding instructions you developed for each entity; or entity extraction can be crowdsourced to citizen scientists.

No masking will be performed.

### **Extraction reliability ***

For each extraction round, list the number of extractors and the procedure used to ensure independent extraction (this can also mean that you declare that you use one extractor, or will not implement procedures to ensure that the extractions are conducted independently). Also explain the test you will use, if any, to assess extractor agreement.

A double data extraction will be conducted by two extractors.

### **Extraction reconciliation procedure ***

For each extraction round, describe the procedure to deal with divergent extraction decisions (if applicable, i.e., if you use more than one extractor).

Any discrepancies and uncertainties that arise will be resolved by discussions with another third reviewer/ among the two extractors.

### **Extraction procedure justification ***

Extraction procedures are often compromises, balancing pragmatic considerations with scientific rigour. Here, describe the justifications for your decisions about the justification of each entity that will be extracted, the extraction rounds, reliability assurance, and reconciliation procedures.

The data extracted are directly relevant to the objectives of the scoping review. Extraction will be done in structured rounds to ensure clarity and consistency. Reliability will be assured through independent verification, and any discrepancies will be resolved through discussion and consensus.

### **Data management and sharing ***

Describe whether and how you will share the files with the extracted entities (as specified in the corresponding field above; i.e., everything extracted from every source, including metadata, method characteristics, variables, associations, etc). List both the file format (e.g., CSV, XLSX, RData), the repository, and any potential embargos or conditions for access. Describe efforts made to share FAIR, 5-star open data, if any such efforts will be made.

Data will be managed in REDCap.

### **Miscellaneous extraction details ***

Here, you can describe any details that are not captured in the other fields in this section.

## Synthesis and Quality Assessment

In this section, you register the procedure for the review’s synthesis: the procedure you designed to use the data that was extracted from each source to answer your research question(s). This often includes transforming the raw extracted data, verifying validity, applying predefined inference criteria, interpreting results, and presenting results. Additionally, you register procedures you designed to assess bias in individual sources and the synthesis itself.

### **Planned data transformations ***

Describe your plans for transforming the raw extracted data. This may include converting effect sizes to other metrics (e.g., convert all metrics to Pearson correlation coefficients); recoding or (re)categorizing extracted qualitative data fragments (e.g., coding extracted music genres within an existing taxonomy); and aggregating extracted data prior to the main synthesis procedures (e.g., compute the mean of a variable over all samples in one source). Applying these transformations to the raw extracted entities from the Extraction section should yield data that corresponds to the variables of interest listed in the Review Methods section.

Not applicable.

### **Missing data ***

Describe how you will deal with missing data (i.e., cases where it is not possible to extract one or more entities from the source material, and your efforts to obtain the missing information, for example by contacting the authors, are not fruitful).

Numbers and percentages will be reported for any missing data.

### **Data validation ***

Describe your process of ensuring that the data are correct and useful (e.g., identifying outliers, identifying retractions, or triangulating with other sources). Also describe your criteria for assessing data validity and how you will deal with data violating those criteria.

Not applicable

**Quality assessment ***

Describe the analyses you plan to do to assess and weigh the quality of the included sources with respect to your research question(s). Examples of tools to use for quality evaluation are Cochrane’s Risk of Bias tool, GRADE, and GRADE-CERQual.

We are assessing the use of estimand framework, the types of intercurrent events, and the corresponding strategies for addressing intercurrent events in anaesthesia clinical trials. Therefore, an assessment of risk of bias/quality will not be included in this review.

**Synthesis plan ***

Describe the specific procedure you will apply to arrive at an answer to the research question(s). For example, in meta-analyses this is the full analysis plan, including any planned subgroup analyses and moderator analyses, the (multilevel) model specification, and preferably the analysis code. For a qualitative review, it is the procedure you plan to use to collate your results into a coherent picture. If you distinguish synthesis tiers (e.g., primary and secondary analysis, or confirmatory and exploratory analyses), list them and indicate which procedures you plan to use for each. Also specify what you will do if parts of the plan can’t be properly executed.

### The results of this review will be documented following the Preferred Reporting Items for Systematic Reviews and Meta-Analyses extension for Scoping Reviews (PRISMA-ScR) guidelines ^9^. Data cleaning and analysis will be conducted using the statistical software R and we will employ descriptive statistics to summarise the data, which will be displayed in various figures and tables. The relationship between the findings and the objectives and questions of the review will be detailed in a narrative format.

**Criteria for conclusions / inference criteria ***

If you plan to draw your conclusions based on pre-specified criteria (e.g., a minimal effect size of interest, a significance level, or a saturation point), list these here.

Not applicable

**Synthesist blinding ***

Describe the procedure, if any, used to blind synthesists (i.e., the persons synthesizing the extracted data to arrive at answers to your research question(s)) from the research questions, hypotheses, and/or specific roles of each extracted entity/variable in this review. For example, for meta-analyses, an analyst external to the main research team can be engaged to perform the analyses without knowing the study’s hypotheses. For qualitative reviews, for the synthesis, other researchers can be involved who are unaware of and are not informed about the research process and expectations.

Not applicable

### **Synthesis reliability ***

List the number of synthesists and the procedure used to ensure independent synthesis (this can also mean that you declare that you use one synthesist, or will not implement procedures to ensure that the syntheses are conducted independently).

Not applicable

### **Synthesis reconciliation procedure ***

Describe the procedure to deal with divergent synthesis decisions (if applicable).

Not applicable

### **Publication bias analyses ***

Describe the analyses you plan to do to assess publication bias (if any). For an overview of commonly used publication bias correction methods, see Table 1 in https://journals.plos.org/plosone/article?id=10.1371/journal.pone.0215052

Not applicable

**Sensitivity analyses / robustness checks ***

Describe the sensitivity analyses or robustness checks you plan to conduct (if any).

Not applicable

**Synthesis procedure justification ***

Extraction procedures are sometimes compromises, balancing pragmatic considerations with scientific rigour. Here, describe the justifications for your decisions about your planned transformations (e.g., if based on assumptions, how do you know those are feasible), your data integrity and missing data checks and corrections, your synthesis plan, the criteria you chose to drive your conclusions/inferences (if any), and your procedures for blinding, and reliability assurance/reconciliation if you use multiple synthesists.

Not applicable

### **Synthesis data management and sharing ***

Describe whether and how you will share the files with the analysis scripts, notes, and outputs. List both the file format (e.g., R scripts, RMarkdown files, plain text files, Open Document files), the repository, and any potential embargos or conditions for access. See https://osf.io/5nk92 for a generic example of an analysis script.

R scripts will be shared upon request.

### **Miscellaneous synthesis details ***

Here, you can describe any details that are not captured in the other fields in this section.

Not Applicable

References

1.	Prisma extension for scoping reviews (PRISMA-ScR): Checklist and explanation. *Ann Intern Med* 2018; **169**:467-73

2.	Goulding KR, Peyton PJ, Story DA, Parker A, Leslie K. Survey of research capability at Australian and New Zealand College of Anaesthetists accredited training sites. *Anaesth Intensive Care* 2017; **45**:196-201

3.	De Silva AP, Leslie K, Braat S, Grobler AC. Application of the estimand framework to anesthesia trials. *Anesthesiology* 2024; **141**:13-23

4.	Darken P, Nyberg J, Ballal S, Wright D. The attributable estimand: A new approach to account for intercurrent events. *Pharm Stat* 2020; **19**:626-35

5.	European Medicines Agency. ICH E9 (R1) addendum on estimands and sensitivity analysis in clinical trials to the guideline on statistical principles for clinical trials. 2020. Available from: <https://www.ema.europa.eu/en/documents/scientific-guideline/ich-e9-r1-addendum-estimands-sensitivity-analysis-clinical-trials-guideline-statistical-principles_en.pdf>

6.	Cro S, Kahan BC, Rehal S*, et al.* Evaluating how clear the questions being investigated in randomised trials are: Systematic review of estimands. *BMJ* 2022; **378**:e070146

7.	Harris PA, Taylor R, Minor BL*, et al.* The REDCap consortium: Building an international community of software platform partners. *J Biomed Inform* 2019; **95**:103208

8.	Harris PA, Taylor R, Thielke R*, et al.* Research electronic data capture (REDCap)—A metadata-driven methodology and workflow process for providing translational research informatics support. *J Biomed Inform* 2009; **42**:377-81

9.	Tricco AC LE, Zarin W, O'Brien KK, Colquhoun H, Levac D, Moher D, Peters MDJ, Horsley T, Weeks L, et al. Prisma extension for scoping reviews (PRISMA-ScR): Checklist and explanation. *Ann Intern Med* 2018; **169**:467-73
