# Supplementary material for: Defining estimands in anaesthesia clinical trials: a scoping review and framework
Source: Br J Anaesth. 2026 Apr 20;137(2):610–24. doi: 10.1016/j.bja.2026.03.026 (PMC13494091; doi:10.1016/j.bja.2026.03.026)
Supplement: Supplementary file 2 [file mmc3.docx]

## Supplementary Text S1. Inclusion and exclusion criteria

Inclusion Criteria

- Published in English Language
- Published between 1/1/2020 - 30/6/2024
- Randomised controlled trials
- Human participants
- Phase 3 or 4 clinical trials
- Clinical trials involving patients who received anaesthesia prior to surgery
- Sample size greater than or equal to 100 patients
- Superiority trials, non-inferiority trials and equivalence trials
- Parallel group, crossover, factorial, individually randomised and cluster randomised designs
- Primary trial results published in:
  - Annals of Internal Medicine
  - British Medical Journal (BMJ)
  - Journal of the American Medical Association (JAMA)
  - The Lancet
  - New England Journal of Medicine (NEJM)
  - PLOS Medicine
  - British Journal of Anaesthesia
  - Anaesthesia
  - Anesthesiology
  - Anesthesia and Analgesia

Exclusion Criteria

- Published before or after the inclusion period
- Published in any language other than English
- Non-randomised trials
- Studies focused on pain medicine for post-operative pain management or chronic pain
- Clinical trials involving gastrointestinal procedures that are diagnostic or minimally invasive
- Sample size less than 100 patients
- Pilot studies and feasibility studies
- Reviews, meta-analyses, sub studies, editorials, or letters
- Interim analyses
- Trials in animals
- Phase 1 and 2 trials
