# Supplementary material for: Defining estimands in anaesthesia clinical trials: a scoping review and framework
Source: Br J Anaesth. 2026 Apr 20;137(2):610–24. doi: 10.1016/j.bja.2026.03.026 (PMC13494091; doi:10.1016/j.bja.2026.03.026)
Supplement: Supplementary file 3 [file mmc4.docx]

## Supplementary Text S2. Full electronic database search strategies

#### Initial Search (carried out on 16/08/2024)

| #1 | (("anesthetics"[MeSH Terms] OR "Anesthesia"[MeSH Terms] OR "anesthe*"[Title] OR "anaesthe*"[Title] OR "anesthe*"[Other Term] OR "anaesthe*"[Other Term] OR "estimand*"[All Fields]) |
| --- | --- |
| #2 | (("clinical trial, phase ii"[Publication Type] OR "clinical trial, phase iii"[Publication Type] OR "clinical trial, phase iv"[Publication Type] OR "Controlled Clinical Trial"[Publication Type] OR "Randomized Controlled Trial"[Publication Type] OR ("clinical trial"[Title] OR "Randomized Controlled Trial"[Title] OR "Randomised Controlled Trial"[Title] OR "Randomised Trial"[Title] OR "Randomized Trial"[Title] OR "Controlled Trial"[Title])) |
| #3 | #1 AND #2 |
| #4 | ("animals"[MeSH Terms] NOT "humans"[MeSH Terms]) |
| #5 | #3 NOT #4 |
| #6 | 2020/01/01:2024/12/31 |
| #7 | #5 AND #6 |

#### Final Search (carried out in 15/10/2024)

Medline (Medical journals)

| #1 | exp controlled clinical trial/ |
| --- | --- |
| #2 | Clinical Trial, Phase III/ or Clinical Trial, Phase IV/ or Multicenter Study/ |
| #3 | (controlled trial or randomised trial or randomized trial or clinical trial).ti. |
| #4 | #1 or #2 or #3 |
| #5 | exp anesthesia/ or exp anesthetic agent/ |
| #6 | exp analgesia/ |
| #7 | airway management/ or exp respiration, artificial/ |
| #8 | (anesthe* or anaesthe* or airway management or airway control or breathing control or breathing management or artificial respiration or artificial ventilat* or pain or analges*).ti,kf. |
| #9 | estimand.mp. |
| #10 | #5 or #6 or #7 or #8 or #9 |
| #11 | #4 and #10 |
| #12 | lancet.so. |
| #13 | new england journal of medicine.so. |
| #14 | annals of internal medicine.so. |
| #15 | 0098-7484.is. |
| #16 | 1756-1833.is. |
| #17 | plos medicine public library of science.jn. |
| #18 | #12 or #13 or #14 or #15 or #16 or #17 |
| #19 | #11 and #18 |
| #20 | limit #19 to yr="2020 -2024" |

Medline (Anaesthesia journals)

| #1 | exp controlled clinical trial/ |
| --- | --- |
| #2 | Clinical Trial, Phase III/ or Clinical Trial, Phase IV/ or Multicenter Study/ |
| #3 | (controlled trial or randomised trial or randomized trial or clinical trial).ti. |
| #4 | #1 or #2 or #3 |
| #5 | 0007-0912.is. |
| #6 | 0003-2409.is. |
| #7 | 0003-3022.is. |
| #8 | 0003-2999.is. |
| #9 | #5 or #6 or #7 or #8 |
| #10 | #4 and #9 |
| #11 | limit #10 to yr="2020 - 2024" |

Ovid EMBASE (Medical Journals)

| #1 | exp controlled clinical trial/ |
| --- | --- |
| #2 | phase 3 clinical trial/ or phase 4 clinical trial/ or multicenter trial/ |
| #3 | (controlled trial or randomised trial or randomized trial or clinical trial).ti. |
| #4 | #1 or #2 or #3 |
| #5 | exp anesthesia/ or exp anesthetic agent/ |
| #6 | exp analgesia/ |
| #7 | respiration control/ or exp artificial ventilation/ or exp assisted ventilation/ |
| #8 | (anesthe* or anaesthe* or airway management or airway control or breathing control or breathing management or artificial respiration or artificial ventilat* or analges*).ti,kf. |
| #9 | estimand.mp. |
| #10 | #5 or #6 or #7 or #8 or #9 |
| #11 | #4 and #10 |
| #12 | lancet.so. |
| #13 | new england journal of medicine.so. |
| #14 | annals of internal medicine.so. |
| #15 | 1549-1277.is. |
| #16 | 0098-7484.is. |
| #17 | 1756-1833.is. |
| #18 | #12 or #13 or #14 or #15 or #16 or #17 |
| #19 | #11 and #18 |
| #20 | limit #19 to yr="2020 - 2024" |

Ovid Embase (Anaesthesia Journals)

| #1 | exp controlled clinical trial/ |
| --- | --- |
| #2 | phase 3 clinical trial/ or phase 4 clinical trial/ or multicenter trial/ |
| #3 | (controlled trial or randomised trial or randomized trial or clinical trial).ti. |
| #4 | #1 or #2 or #3 |
| #5 | 0003-2409.is. |
| #6 | 0007-0912.is. |
| #7 | 0003-3022.is. |
| #8 | 0003-2999.is. |
| #9 | #5 or #6 or #7 or #8 |
| #10 | #4 and #9 |
| #11 | limit #10 to yr="2020 - 2024" |

Cochrane Database of Systematic Reviews (For Medical Journals)

| #1 | exp anesthesia/ or exp anesthetic agent/ |
| --- | --- |
| #2 | exp analgesia/ |
| #3 | airway management/ or exp respiration, artificial/ |
| #4 | (anesthe* or anaesthe* or airway management or airway control or breathing control or breathing management or artificial respiration or artificial ventilat* or analges*).ti,kf. |
| #5 | estimand.mp. |
| #6 | #1 or #2 or #3 or #4 or #5 |
| #7 | Lancet.so. |
| #8 | new england journal of medicine.so. |
| #9 | annals of internal medicine.so. |
| #10 | 0098-7484.is. |
| #11 | 1549-1277.is. |
| #12 | 0959-8138.is. |
| #13 | #7 or #8 or #9 or #10 or #11 or #12 |
| #14 | #6 and #13 |
| #15 | limit #14 to yr="2020 - 2024" |

Cochrane Database of Systematic Reviews (For Anaesthesia Journals)

| #1 | 0003-2409.is. |
| --- | --- |
| #2 | 0007-0912.is. |
| #3 | 0003-3022.is. |
| #4 | 0003-2999.is. |
| #5 | #1 or #2 or #3 or #4 |
| #6 | limit #5 to yr="2020 - 2024" |
