# Supplementary material for: Defining estimands in anaesthesia clinical trials: a scoping review and framework
Source: Br J Anaesth. 2026 Apr 20;137(2):610–24. doi: 10.1016/j.bja.2026.03.026 (PMC13494091; doi:10.1016/j.bja.2026.03.026)
Supplement: Supplementary file 4 [file mmc5.docx]

## Supplementary Figure S1. PRISMA 2020 flow diagram for study selection

**Identification**

Abstract screened **(n = 1684)**

Full text screened **(n = 240)**

References removed in Covidence **(n = 18)**

Duplicates identified manually (n = 11)

Duplicates identified by Covidence (n = 7)

Studies not eligible (**n = 1444)**

Studies excluded **(n = 101)**

Available abstract only (n = 44)

Dose–response study (n = 1)

Duplicate record (n = 1)

No surgery involved (n = 16)

Non-relevant journal (n = 1)

Non-relevant outcome (n = 2)

Not anaesthesia-related (n = 2)

Not a randomised controlled trial (n = 14)

Phase 2 study (n=1)

Published outside time frame (n = 12)

Sample size <100 (n = 3)

Sub-study of parent trial (n = 4)

**Included**

Studies included in the review **(n = 139)**

**Screening**

Studies from databases **(n = 4670)**

MEDLINE (n = 177)

Embase (n = 2379)

Cochrane (n = 2114)

Duplicates removed in Endnote **(n = 2968)**

Fig. 1. Preferred Reporting Items for Systematic Reviews and Meta-Analyses (PRISMA) flow diagram
