# Supplementary material for: Defining estimands in anaesthesia clinical trials: a scoping review and framework
Source: Br J Anaesth. 2026 Apr 20;137(2):610–24. doi: 10.1016/j.bja.2026.03.026 (PMC13494091; doi:10.1016/j.bja.2026.03.026)
Supplement: Supplementary file 5 [file mmc6.docx]

## Supplementary Figure S2. Framework Navigator

To illustrate the framework’s application, consider a researcher planning a trial comparing Positive End-Expiratory Pressure (PEEP) versus Zero End-Expiratory Pressure (ZEEP) during general anaesthesia, with postoperative pulmonary complications as the primary outcome. Using our tool, they would first select the main intervention category “General anaesthesia”, followed by the relevant subcategories “Airway management” and “Ventilator settings.” They would then choose the corresponding outcome domain from the Standardised Endpoints in Perioperative Medicine (StEP) framework ^1-10^ (e.g., “Pulmonary complications”). Based on these selections, the tool automatically displays potential intercurrent events commonly encountered in anaesthesia trials for this specific intervention–outcome combination, drawn from the trials included in our scoping review. This provides clinicians and trialists with an immediate, evidence-informed starting point to discuss intercurrent events, estimand definitions and appropriate intercurrent event-handling strategies.

The steps below outline this process in a generalised, user-friendly format that can be applied to any intervention–outcome combination.

## **Step 1: Choose the intervention**

Begin by selecting the main intervention category from the drop-down menu. Where applicable, choose one or more subcategories that best describe your intervention. To assist you in this process, the adjacent column provides example interventions from our scoping review for each category. These examples can help you determine which category, or subcategory best matches your intervention.

| **Framework Navigator** | | | |
| --- | --- | --- | --- |
| **Intervention selection** | | **Example interventions in the chosen combination** | **Potential intercurrent events** |
| **Select intervention main category** | General anaesthesia | Ventilation mode  Individualised open-lung approach  Mechanical ventilation  Fully automated ventilation  Driving pressure-guided ventilation vs conventional protective ventilation PEEP   |  |
| **Select intervention subcategory 1**  **(if applicable)** | Airway management |  |  |
| **Select intervention subcategory 2**  **(if applicable)** | Ventilator settings |  |  |
|  | |  |  |
| **Primary outcome selection** | | **Example outcomes in this domain** |  |
|  | |  |  |

**Step 2: Choose the outcome**

Next, select the primary outcome category that best reflects your trial’s main endpoint. Outcomes are organised according to the StEP framework ^1-10^, which groups endpoints into domains such as mortality, morbidity, pulmonary, cardiovascular, renal, and patient comfort outcomes. For outcomes not covered by StEP domains, select the “Other” category (e.g., first-attempt success rate). The adjacent column provides example outcomes from our scoping review to help you identify the most appropriate outcome category for your research question.

| **Framework Navigator** | | | |
| --- | --- | --- | --- |
| **Intervention selection** | | **Example interventions in the chosen combination** | **Potential intercurrent events** |
| **Select intervention main category** | General anaesthesia | Ventilation mode  Individualised open-lung approach  Mechanical ventilation  Fully automated ventilation  Driving pressure-guided ventilation vs conventional protective ventilation PEEP |  |
| **Select intervention subcategory 1**  **(if applicable)** | Airway management |  |  |
| **Select intervention subcategory 2**  **(if applicable)** | Ventilator settings |  |  |
| **Primary outcome selection** | | **Example outcomes in this domain** |  |
| **Select primary outcome domain** | Pulmonary complications | Postoperative pulmonary complications |  |
|  | |  |  |

**Step 3: Explore the mapped intercurrent events**

Once both the intervention and outcome categories have been selected, the framework automatically displays the potential intercurrent events most frequently reported for that specific intervention–outcome combination in our scoping review. Users can review these potential intercurrent events to anticipate which events may arise in their study and to consider appropriate handling strategies when defining estimands.

| **Framework Navigator** | | | |
| --- | --- | --- | --- |
| **Intervention selection** | | **Example interventions in the chosen combination** | **Potential intercurrent events** |
| **Select intervention main category** | General anaesthesia | Ventilation mode  Individualised open-lung approach  Mechanical ventilation  Fully automated ventilation  Driving pressure-guided ventilation vs conventional protective ventilation PEEP | Protocol deviations  Use of drugs to correct hypotension  Use of rescue medication  Supportive/additional airway adjuncts  Pulmonary embolism  Treatment discontinuation  Surgery cancellations  Hypoxia  Failure to initiate the randomised treatment  Death  Acute kidney injury   |
| **Select intervention subcategory 1**  **(if applicable)** | Airway management |  |  |
| **Select intervention subcategory 2**  **(if applicable)** | Ventilator settings |  |  |
|  | |  |  |
| **Primary outcome selection** | | **Example outcomes in this domain** |  |
| **Select primary outcome domain** | Pulmonary complications | Postoperative pulmonary complications |  |
|  | |  |  |

**References**

1.	Beattie WS, Lalu M, Bocock M*, et al.* Systematic review and consensus definitions for the standardized endpoints in perioperative medicine (StEP) initiative: Cardiovascular outcomes. *Br J Anaesth* 2021; **126**:56-66

2.	Myles PS, Boney O, Botti M*, et al.* Systematic review and consensus definitions for the standardised endpoints in perioperative medicine (StEP) initiative: Patient comfort. *Br J Anaesth* 2018; **120**:705-11

3.	McIlroy DR, Bellomo R, Billings FTI*, et al.* Systematic review and consensus definitions for the standardised endpoints in perioperative medicine (StEP) initiative: Renal endpoints. *Br J Anaesth* 2018; **121**:1013-24

4.	Buggy DJ, Freeman J, Johnson MZ*, et al.* Systematic review and consensus definitions for standardised endpoints in perioperative medicine: Postoperative cancer outcomes. *Br J Anaesth* 2018; **121**:38-44

5.	Haller G, Bampoe S, Cook T*, et al.* Systematic review and consensus definitions for the standardised endpoints in perioperative medicine initiative: Clinical indicators. *Br J Anaesth* 2019; **123**:228-37

6.	Barnes J, Hunter J, Harris S*, et al.* Systematic review and consensus definitions for the standardised endpoints in perioperative medicine (StEP) initiative: Infection and sepsis. *Br J Anaesth* 2019; **122**:500-8

7.	Jackson AIR, Boney O, Pearse RM*, et al.* Systematic reviews and consensus definitions for the standardised endpoints in perioperative medicine (StEP) initiative: Mortality, morbidity, and organ failure. *Br J Anaesth* 2023; **130**:404-11

8.	Myles PS, Wallace S, Boney O*, et al.* An updated systematic review and consensus definitions for standardised endpoints in perioperative medicine: Patient comfort and pain relief. *Br J Anaesth* 2025; **134**:1450-9

9.	Moonesinghe SR, Jackson AIR, Boney O*, et al.* Systematic review and consensus definitions for the standardised endpoints in perioperative medicine initiative: Patient-centred outcomes. *Br J Anaesth* 2019; **123**:664-70

10.	Abbott TEF, Fowler AJ, Pelosi P*, et al.* A systematic review and consensus definitions for standardised end-points in perioperative medicine: Pulmonary complications. *Br J Anaesth* 2018; **120**:1066-79
