# Supplementary material for: Defining estimands in anaesthesia clinical trials: a scoping review and framework
Source: Br J Anaesth. 2026 Apr 20;137(2):610–24. doi: 10.1016/j.bja.2026.03.026 (PMC13494091; doi:10.1016/j.bja.2026.03.026)
Supplement: Supplementary file 7 [file mmc9.docx]

## Supplementary Table S3. Characteristics of included trials

| Characteristic | Total, n (%)  N = 139 |
| --- | --- |
| Journal  Anaesthesia  Medical | 126 (91%)  13 (9%) |
| Trial Objective  Superiority  Non-inferiority  Equivalence  Unclear | 120 (86%)  17 (12%)  1 (1%)  1 (1%) |
| Trial design  Parallel group  Crossover  Factorial | 137 (99%)  1 (1%)  1 (1%) |
| Funder Type  Pharmaceutical/ For-Profit  Other (academic, government, or investigator-initiated sources)  Unclear | 9 (6%)  126 (91%)  4 (3%) |
| Reported the trial registration | 138 (99%) |
| Availability of the trial protocol  Publicly available  Available upon request  Not publicly available  Not Mentioned | 53 (38%)  20 (14%)  15 (11%)  51 (37%) |
| Availability of the statistical analysis plan  Available  Not available | 33 (24%)  106 (76%) |
| Stated compliance with CONSORT guideline | 79 (57%) |

CONSORT = Consolidated Standards of Reporting Trials
